# Supplementary material for: A multi-omic characterization of temperature stress in a halotolerant Scenedesmus strain for algal biotechnology
Source: Commun Biol. 2021 Mar 12;4:333. doi: 10.1038/s42003-021-01859-y (PMC7955037; doi:10.1038/s42003-021-01859-y)
Supplement: Supplementary file 2 — Supplementary Information [file 42003_2021_1859_MOESM2_ESM.pdf]

Supplemental Information

A multi-omic characterization of temperature stress in a halotolerant *Scenedesmus* strain for algal biotechnology

Sara Calhoun[1,2*], Tisza Ann Szeremy Bell[3,4*], Lukas R. Dahlin[5*], Yuliya Kunde[3], Kurt LaButti[1], Katherine B. Louie[1], Andrea Kuftin[1], Daniel Treen[1], David Dilworth[1], Sirma Mihaltcheva[1], Christopher Daum[1], Benjamin P. Bowen[1], Trent R. Northen[1], Michael T. Guarnieri[5], Shawn R. Starkenburg[3], Igor V. Grigoriev[1,2,6]

[1]US Department of Energy Joint Genome Institute, Lawrence Berkeley National Laboratory, Berkeley, CA 94720, USA
[2]Environmental Genomics and Systems Biology, Lawrence Berkeley National Laboratory, Berkeley, CA 94720, USA
[3]Applied Genomics Team, Bioscience Division, Los Alamos National Laboratory, Los Alamos, NM 87545, USA
[4]Division of Biological Sciences, Genome Core, University of Montana, Missoula, MT 59801, USA
[5]National Bioenergy Center, National Renewable Energy Laboratory, Golden, CO 80401, USA
[6]Department of Plant and Microbial Biology, University of California Berkeley, Berkeley, CA 94720, USA

Authors contributed to work equally*

Corresponding Authors: Shawn Starkenburg <shawns@lanl.gov> and Igor V. Grigoriev <ivgrigoriev@lbl.gov>

Supplementary Note 1. Description of the chloroplast and mitochondrial genomes

The chloroplast and mitochondrial genomes of *Scenedesmus* sp. NREL 46B-D3 were assembled and annotated in addition to the nuclear genome. The 168,306 bp chloroplast assembly consists of 7 contigs with 69 predicted protein-coding genes, comparable to the *Chlamydomonas reinhardtii*[1] and Sphaeropleales chloroplast genomes (Supplementary Figure 11). The annotated genes include chlorophyll biosynthesis genes (chlB, chlL, chlN), RuBisCO gene, photosystem I/II genes, cytochrome b_6f genes, ATP synthase genes, RNA polymerase genes, and ribosomal protein genes. There are also 11 rRNA genes and 29 tRNA genes predicted in the chloroplast genome, similar to the chloroplast genomes of other Sphaeropleales. The 64,661 bp mitochondrial genome assembly consists of 3 contigs and contains 14 predicted protein-coding, 9 rRNA, and 34 tRNA genes. The number of tRNA genes is larger than other Sphaeropleales mitochondrial genomes, and the assembly is larger than all except the *Monoraphidium neglectum* mitochondrial genome. Otherwise, the number of genes is comparable to the other mitochondrial genomes.

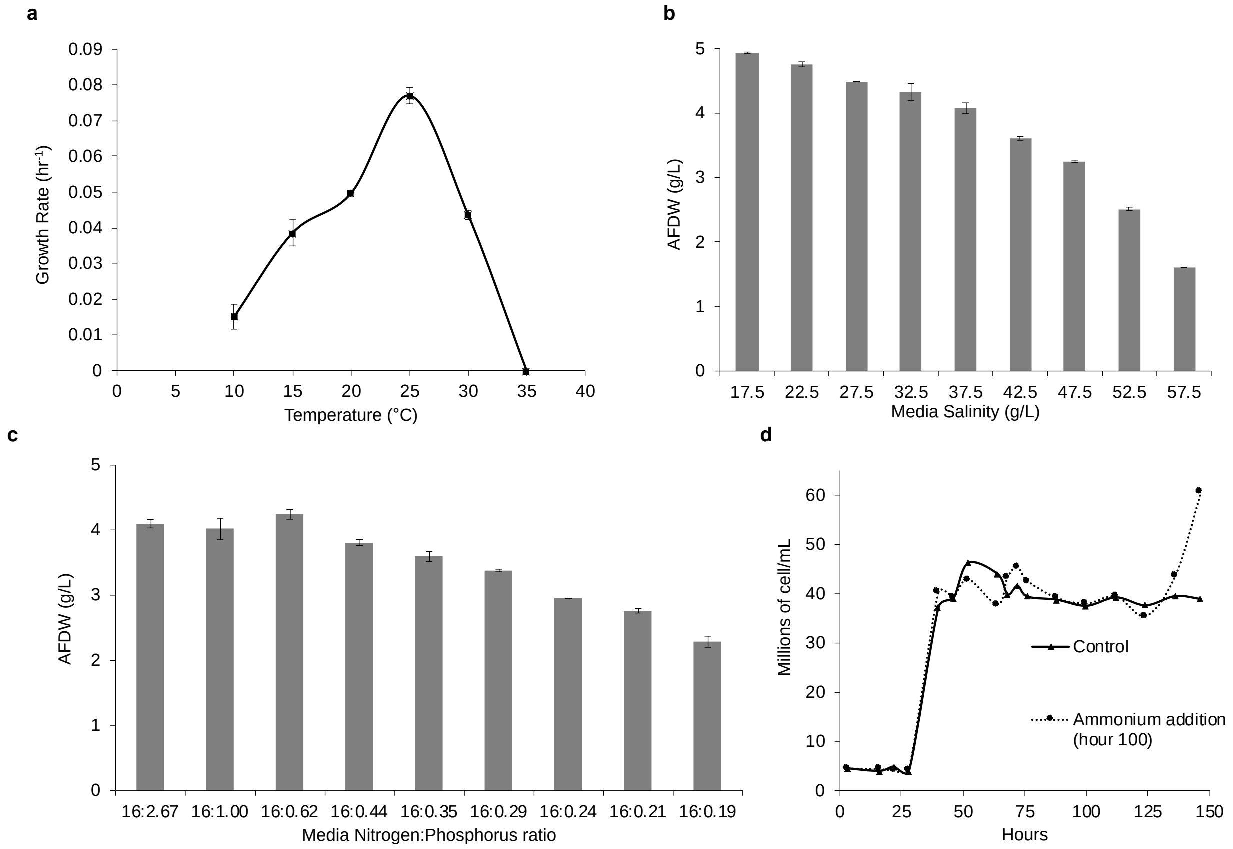

Supplementary Figure 1. Physiological characterization under varied temperature, salinity, and nitrogen regimes. a) Temperature optima of *Scenedesmus* sp. NREL 46B-D3, data points represent the average and standard deviation of n=3 biological replicates. b) Salinity tolerance screening of *Scenedesmus* sp. NREL 46B-D3, data points represent the average and standard deviation of n=2 biological replicates, following 6 days of growth. c) Nitrogen: Phosphorus ratio screening of *Scenedesmus* sp. NREL 46B-D3, data points represent the average and standard deviation of n=2 biological replicates, following 6 days of growth. d) Representative growth curves of *Scenedesmus* sp. NREL 46B-D3. With and without addition of ammonium chloride to a final concentration of 5 mM at hour 100, following entry into stationary growth phase.

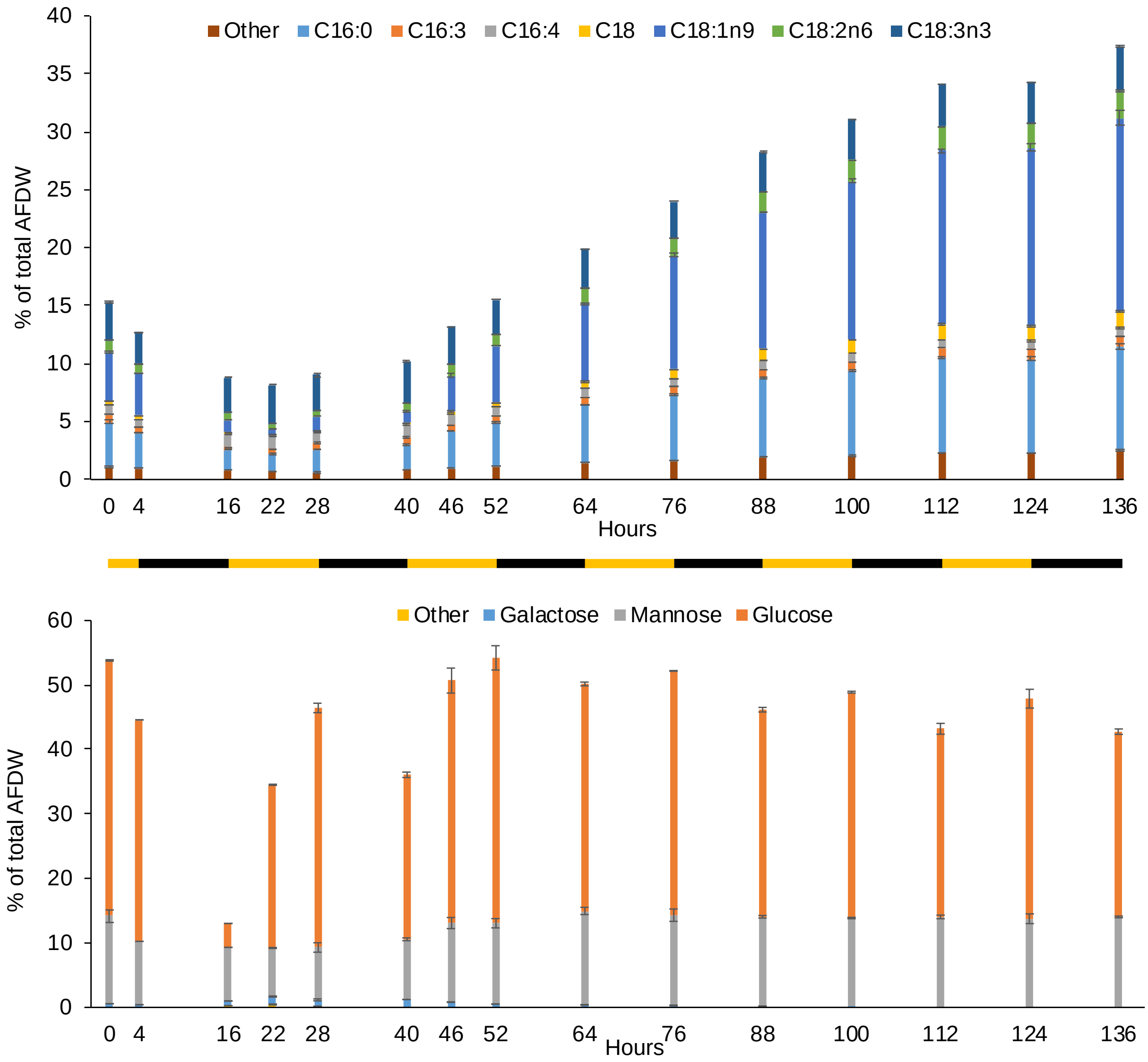

Supplementary Figure 2. Lipid and carbohydrate speciation. Top: Lipid analysis via fatty acid methyl esters as a function of time. Bottom: Carbohydrate analysis via acid hydrolysis of the biomass, as a function of time, all data points are an average of n=2 biological replicates; error bars depict the standard deviation of the replicates. Alternating black and yellow bar represents the lighting cycle.

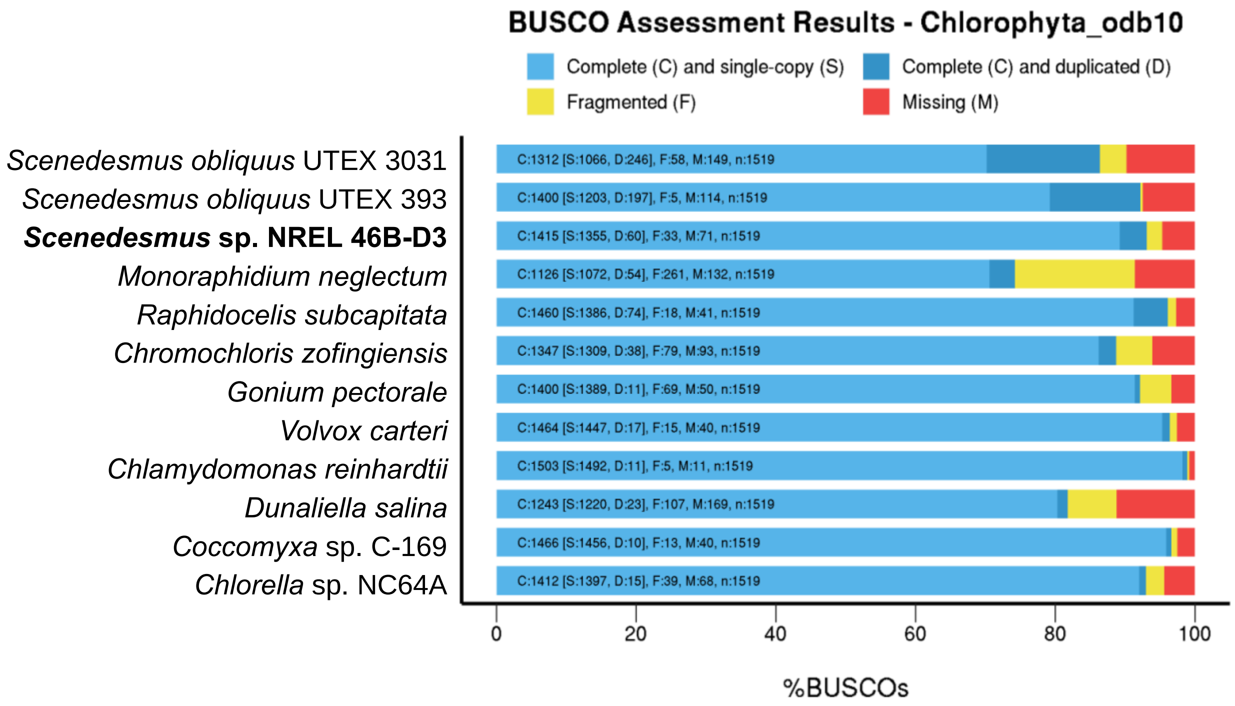

Supplementary Figure 3. Completeness assessed by BUSCO analysis of annotated genomes using the Chlorophyta ortholog set (chlorophyta_odb10; 11-20-19) consisting of 1,519 near-universal single-copy orthologs. For diploid genomes, a single haplotype was used in this comparison.

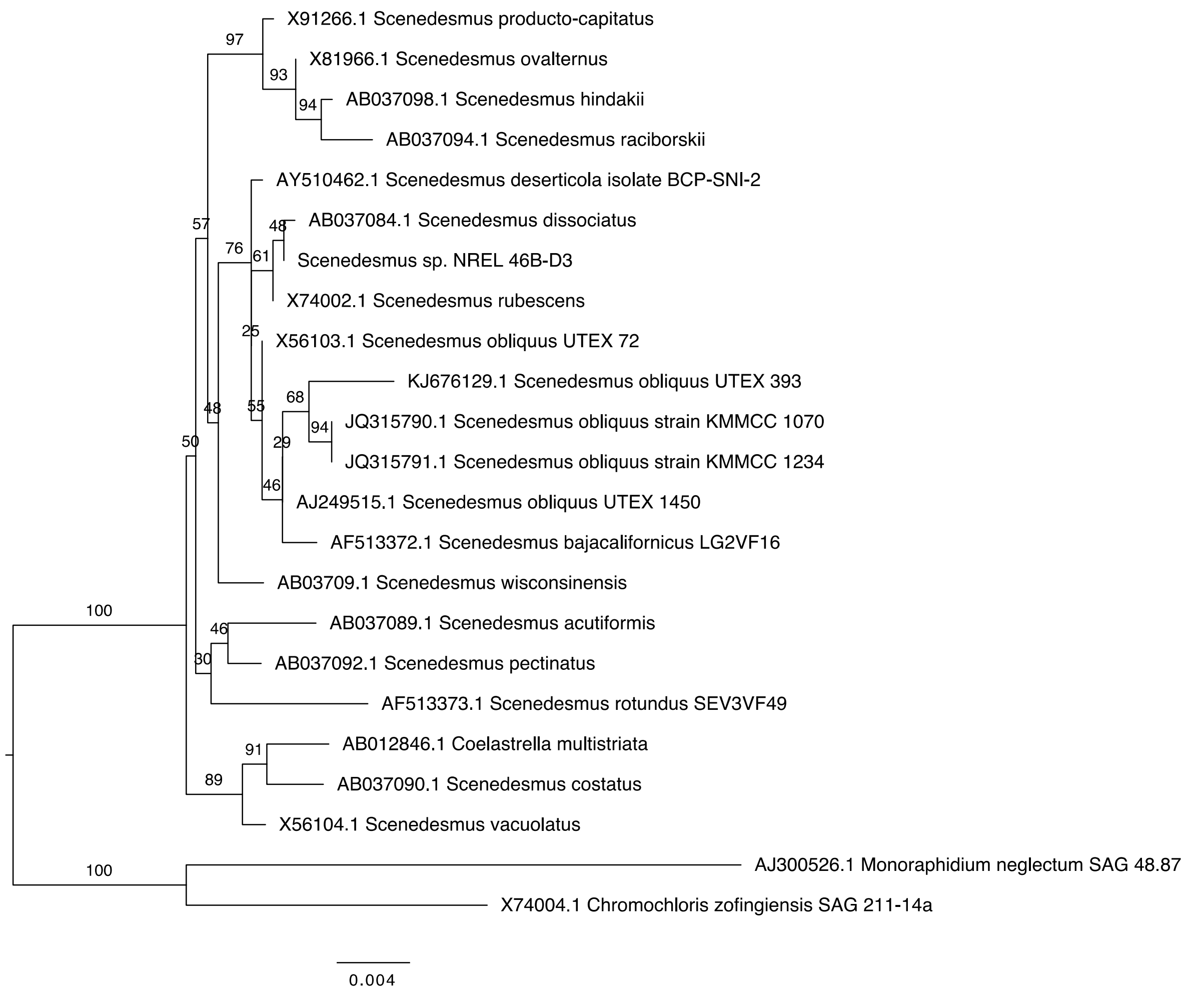

Supplementary Figure 4. Maximum-likelihood 18S rRNA phylogenetic tree of *Scenedesmus* sp. NREL 46B-D3, other *Scenedesmus*-like strains, and outgroups, *Chromochloris zofingiensis* and *Monoraphidium neglectum*. Scale bar shows the mean number of nucleotide substitutions per site.

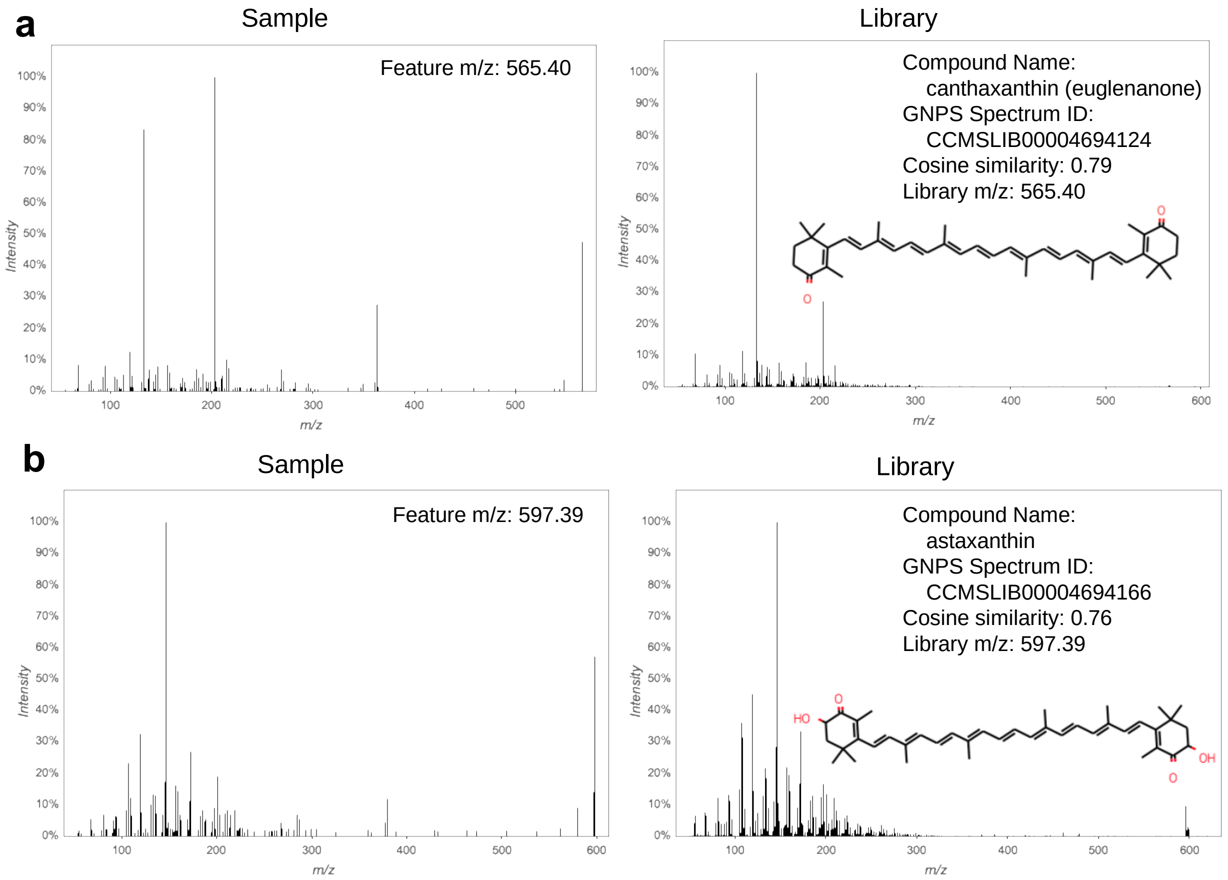

Supplementary Figure 5. Fragmentation spectra for sample features (left) and matching GNPS library compounds (right) for carotenoid pigments canthaxanthin (a) and astaxanthin (b). The spectra for other putative identifications, including beta-cryptoxanthin, can be publicly accessed at https://gnps.ucsd.edu/ProteoSAFe/status.jsp?task=e971a91e4ebd489f851a915c08d19ced for the positive ion mode dataset and https://gnps.ucsd.edu/ProteoSAFe/status.jsp?task=6b7d66e2b1cd487d9d8c5f345435b8f8 for the negative ion mode dataset.

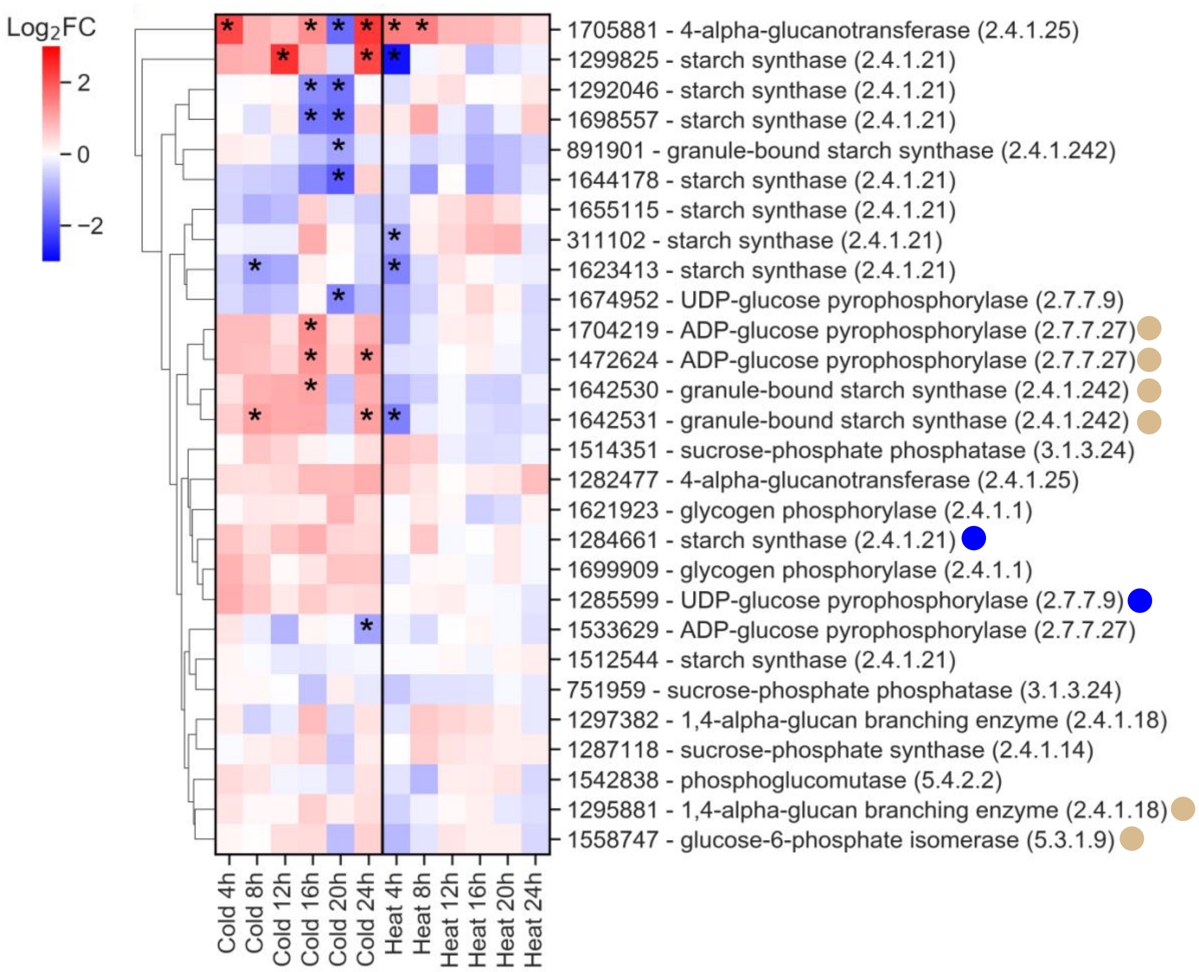

Supplementary Figure 6. Heat map of fold change of starch and sucrose metabolism genes in cold stress and heat stress samples over time. Colored circles next to the genes indicate membership to a co-expression gene module enriched with fatty acid biosynthesis genes and consistent with colors shown in Figure 7. Asterisk (*) indicates p-value < 0.1 and absolute fold change > 1. Rows are ordered by hierarchical clustering of mean fold change over three replicates.

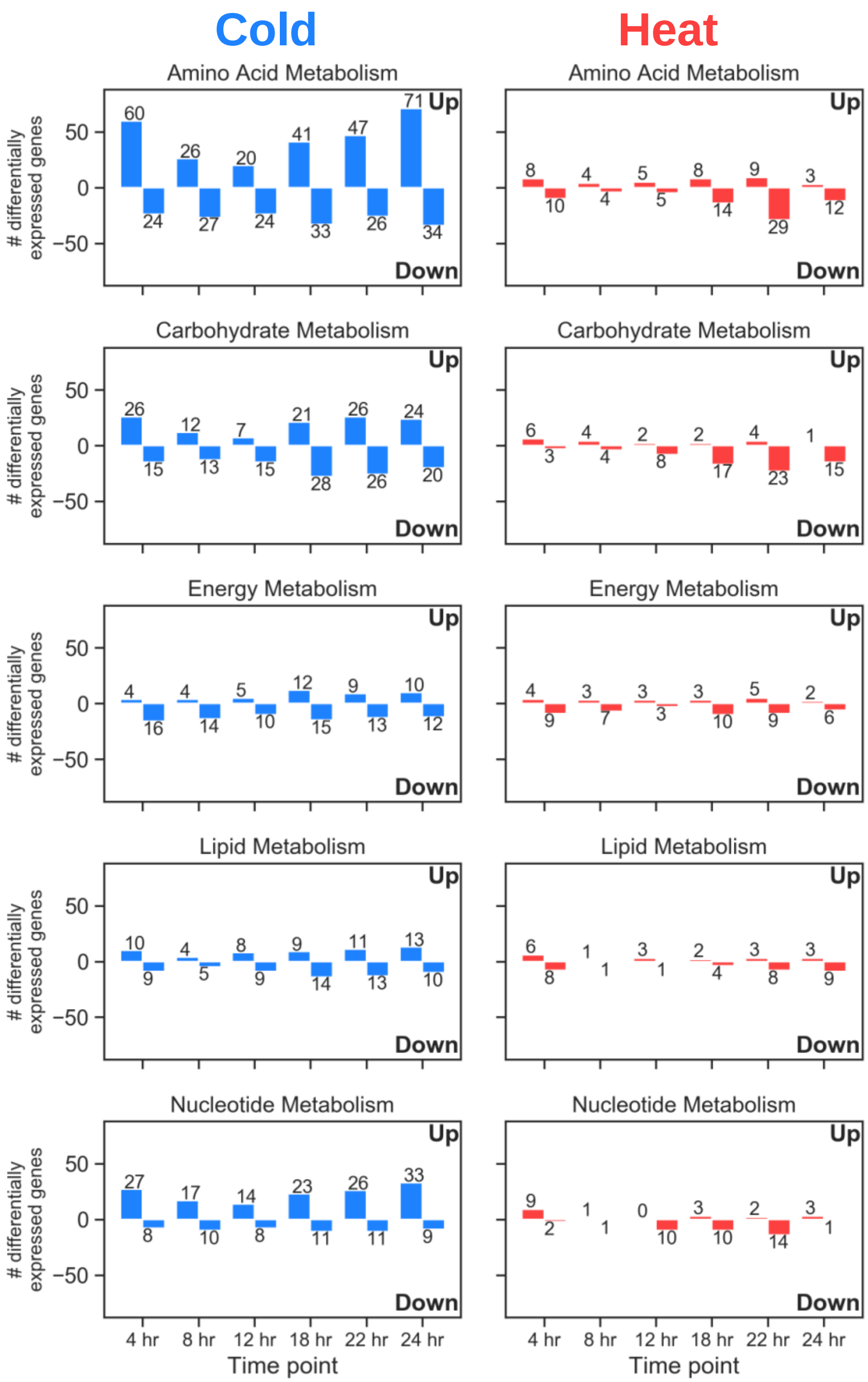

Supplementary Figure 7. Numbers of up and down regulated genes divided by KEGG classification based on differential expression analysis between treatment and control samples with three replicates each.

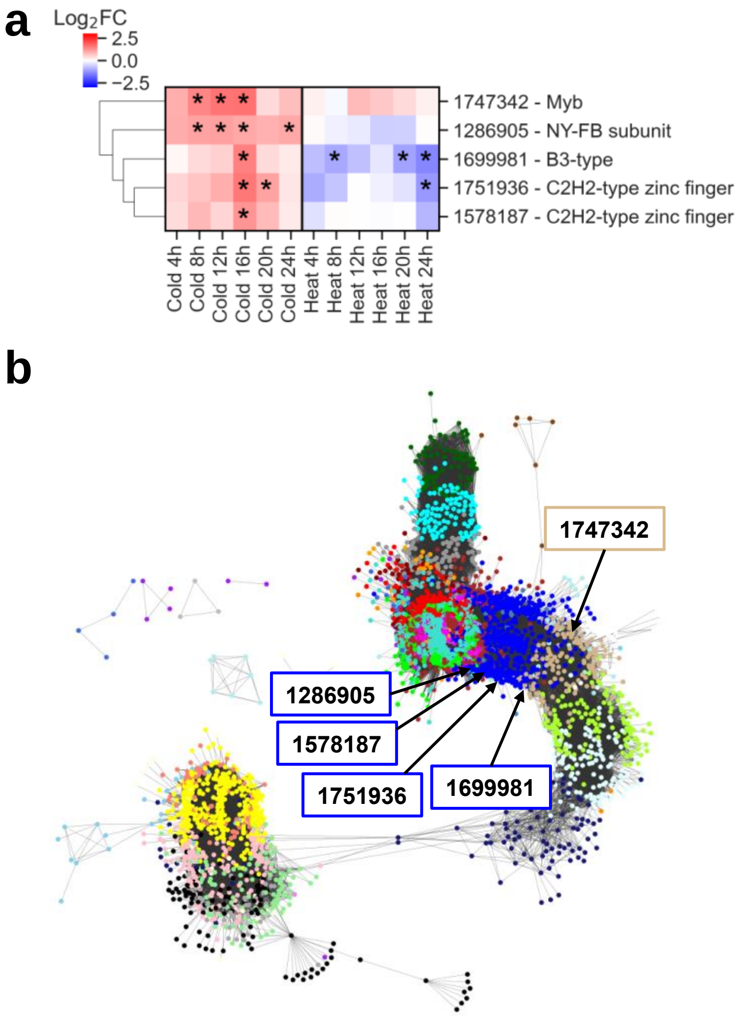

Supplementary Figure 8. Transcription factors increased in cold stress. A) Heat map of fold change for TFs identified by a co-expression module enriched with fatty acid metabolism genes. Asterisk (*) indicates p-value < 0.1 and absolute fold change > 1. Rows are ordered by hierarchical clustering of mean fold change over three replicates. B) Co-expression network constructed by weighted gene co-expression network analysis (WGCNA) with genes colored by clusters (same as Figure 7) with selected TF genes shown. The borders of TF labels are colored by cluster.

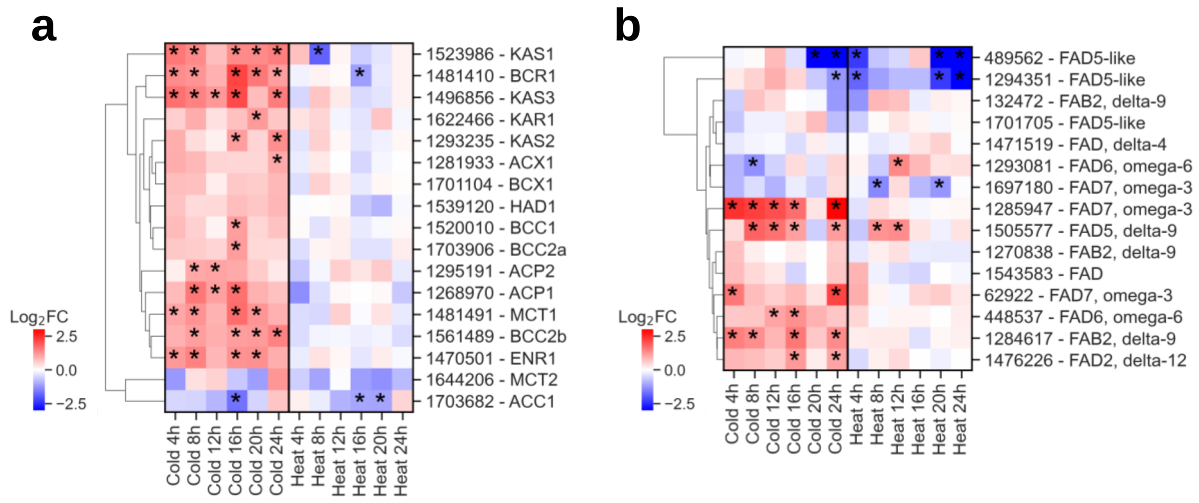

Supplementary Figure 9. Heat map of fold change of (a) fatty acid biosynthesis genes and (b) fatty acid desaturase genes in cold stress and heat stress samples over time. Asterisk (*) indicates p-value < 0.1 and absolute fold change > 1. Rows are ordered by hierarchical clustering of mean fold change over three replicates.

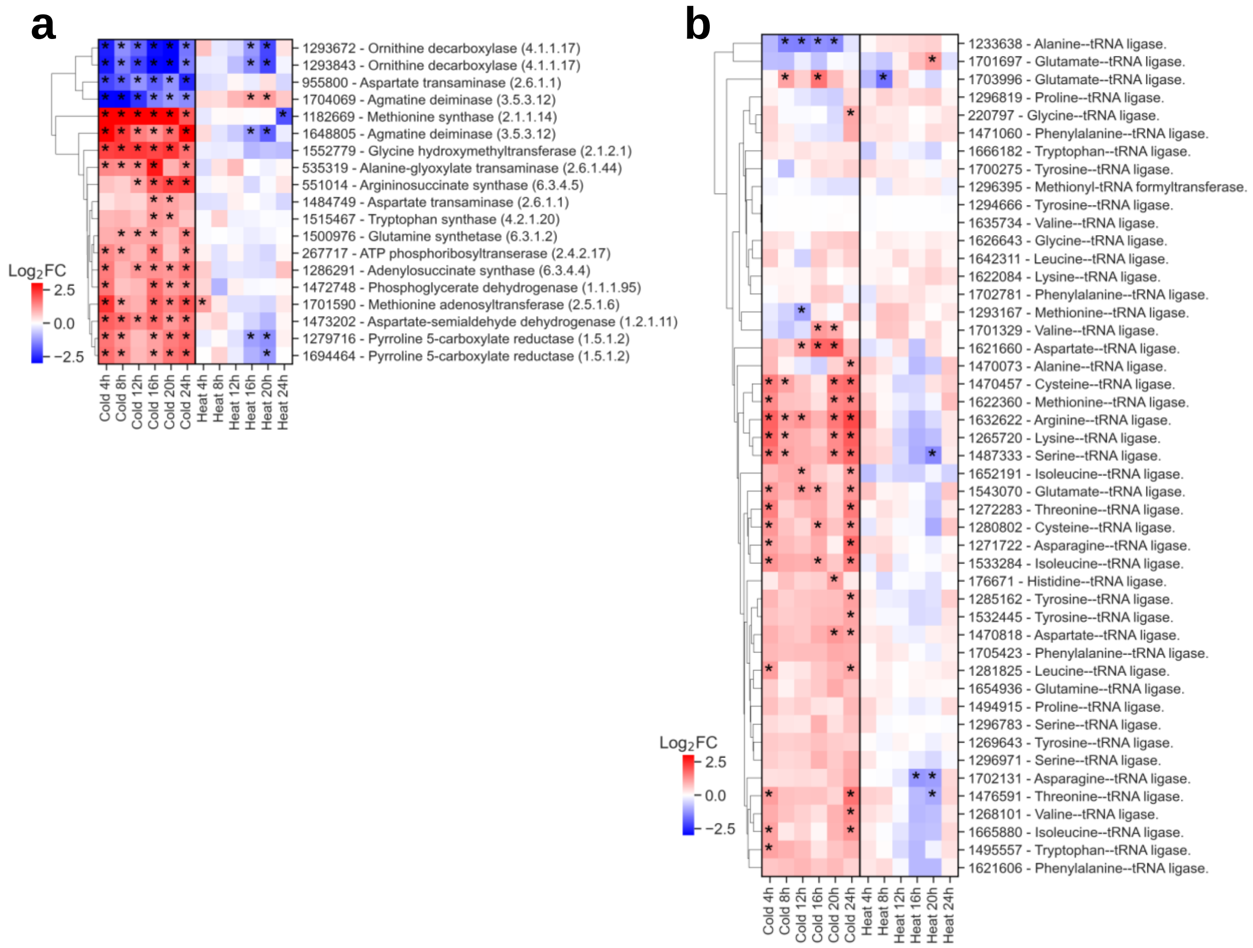

Supplementary Figure 10. Changes in expression of genes involved in amino acid metabolism. a) Heat map of fold change of genes involved in amino acid metabolism. b) Heat map of fold change of representative aminoacyl tRNA ligase genes in cold stress and heat stress samples over time. Asterisk (*) indicates p-value < 0.1 and absolute fold change > 1. Rows are ordered by hierarchical clustering of mean fold change over three replicates.

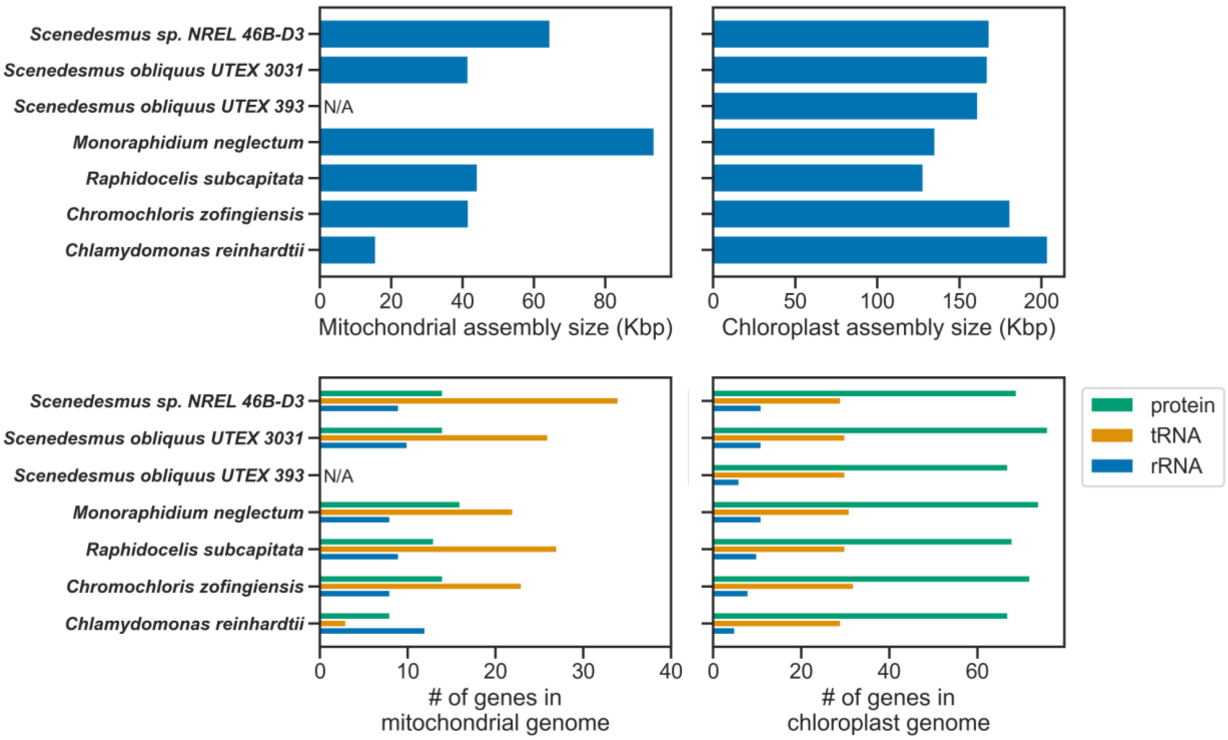

Supplementary Figure 11. Comparison of mitochondrial genomes (left) and chloroplast genomes (right) of Sphaeropleales species and *Chlamydomonas reinhardtii.*

Supplementary Table 1. Assembly and gene prediction statistics for the nuclear draft genome of *Scenedesmus* sp. NREL 46B-D3.

	Scenedesmus sp. NREL 46B-D3	*Scenedesmus obliquus* UTEX 3031[2]	*Scenedesmus (Tetradesmus) obliquus* UTEX 393[3-5]	*Raphidocelis subcapitata* NIES-35[6]	*Monoraphidium neglectum*[7]	*Chromochloris zofingiensis*[8]	*Chlamydomonas reinhardtii*[1,9,10]
Assembly size, Mbp	152	210	108	51	70	60	112
G + C content	57%	56%	57%	72%	65%	51%	64%
# scaffolds/contigs	2,661	2,812	1,368/1,532	300/1,480	6,720/11,901	217	54/1,495
Scaffold/ contig N50	181	348	177/207	46/149	1302/2125	8	7/140
Scaffold/contig L50, Mbp	0.204	0.152	0.187/ 0.16	0.342/ 0.098	0.016/ 0.009	3.27	7.784/ 0.219
# nuclear genes	17,399	19,873	16,779	13,383	16,755	15,274	16,709
Avg. protein length, aa	407	479	593	561	348	482	497
Exons per gene	6.06	7.55	7.05	6.69	5.04	4.97	7.4
Avg. gene length, bp	3,664	4,548	4,022	2,980	2,265	2,504	3,895
Avg. transcript length, bp	1,710	1,945	1,778	1,684	1,045	1,447	1,768
Avg. exon length, bp	282	258	252	252	207	291	240
Avg. intron length, bp	388	400	373	230	304	269	336
Chloroplast genome size, bp	168,306	167,272	161,452	128,080	135,362	181,058	203,828
# Chloroplast genes	69	76	67	68	74	72	67
Mitochondrial genome size, bp	64,661	41,704		44,268	93,840	41,733	15,758
# mitochondrial genes	14	14		13	16	14	8

Supplementary Table 2. List of transcription factor gene families unique to *Scenedesmus* strains.

Gene families unique to NREL 46B-D3 are highlighted in grey.

TF family description	Pfam IDs	Genes in *Scenedesmus* sp. NREL 46B-D3	# of genes		
			Scenedesmus sp. NREL 46B-D3	*S. obliquus* UTEX 3031	*S. obliquus* UTEX 393
**GATA zinc finger**	**PF00320**	**1480750,1480764,1480782,1524290,225802**	**5**	**0**	**0**
**Myb-like domain**	**PF00249**	**1457170,1700284,1704709,884777**	**4**	**0**	**0**
AP2 domain	PF00847	1497086,1696516,664399	3	2	1
Zinc finger C-x8-C-x5-C-x3-H type		1063463,1466585,621515	3	1	0
SBP domain	PF03110	1627375,43674	2	5	3
Basic region leucine zipper	PF07716	1702216,760865	2	0	1
**Leucine Rich repeat**	**PF13516**	**1634233,1646767**	**2**	**0**	**0**
**Transcriptional activator of glycolytic enzymes**	**PF12550**	**1641403,1701388**	**2**	**0**	**0**
AP2 domain	PF00847	1625871	1	1	3
AP2 domain	PF00847	914794	1	2	2
C2H2		1463546	1	2	2
SBP domain	PF03110	706206	1	2	1
AP2 domain	PF00847	1697347	1	1	1
Myb-like domain	PF00249	420997	1	2	0
cysteine-rich polycomb-like protein (CPP)	PF03638	1702932	1	1	1
cysteine-rich polycomb-like protein (CPP)	PF03638	1459954	1	2	0
AP2 domain	PF00847	249012	1	1	1
SBP domain	PF03110	1007664	1	1	1
SBP domain	PF03110	1635426	1	1	1
SBP domain	PF03110	1635691	1	1	1
SBP domain	PF03110	1644723	1	1	0
AP2 domain	PF00847	1646869	1	1	0
C2H2	PF00096	1646871	1	1	0
bZIP	PF00170	192015	1	0	1
**SBP domain**	**PF03110**	**1659981**	**1**	**0**	**0**

**G2-like**		**211467**	**1**	**0**	**0**
**C2H2**		**388996**	**1**	**0**	**0**
**bZIP**		**506544**	**1**	**0**	**0**
**bHLH**		**555816**	**1**	**0**	**0**
**AT hook motif**	**PF02178**	**643985**	**1**	**0**	**0**
**Zinc finger C-x8-C-x5-C-x3-H type**	**PF00642**	**843795**	**1**	**0**	**0**

Supplementary Table 3. List of EC numbers by fatty acid biosynthesis, fatty acid elongation, and glycerolipid metabolism

**Fatty Acid Biosynthesis**	
EC Number	Protein Ids
1.1.1.100	1622466,1537787
1.14.19.2	132472,1270838,1284617
1.3.1.104	347180
1.3.1.9	1470501
2.3.1.180	1496856
2.3.1.39	1644206,1481491
2.3.1.41	1523986,1664879,1293235
3.1.2.14	819689,1561707
4.2.1.59	1539120
6.2.1.3	1702652,1704532,1706112,1621613,1281717,1530236
6.4.1.2	1281933,1701104
**Fatty Acid Elongation**	
EC Number	ProteinIds
1.1.1.330	1488684,1621378
1.1.1.35	227276
1.3.1.93	1285929
2.3.1.16	1703173
2.3.1.199	1643915,1294629,1697728,1272662,1296934,1699688,1271278,1295676,1292456,1623470,1266421,1271769,1663663,1653033,1464191,1292674,1271778,1286152,519050,1285682,1697334,1272325,1219477,1274916,1296845,1634579
3.1.2.22	1266007
4.2.1.134	278611,135824
4.2.1.17	1500938,1076713
**Glycerolipid Metabolism**	
EC Number	ProteinIds
1.1.1.21	1285820,1699035,572243
1.2.1.3	1621753,1284160,811780,1698243,1699593
2.3.1.15	1473792,1526229
2.3.1.158	1530663
2.3.1.20	1697247,1644430,1524035,1638631,1470767,1465599,870958,1706814,1624307

2.3.1.22	17606
2.3.1.51	1697361,1701277
2.4.1.241	1639065,1693088,136069
2.4.1.46	1691478
2.7.1.107	309756,1706007,1703204,1700973
2.7.1.29	924665
2.7.1.30	1268650
2.7.1.31	1700000
2.7.7.9	1674952,1285599
3.1.1.3	1528817,1067358,328337,501826,1151596,1702416,1705545,1286300,54834,1703694,1701491,1705829,378961,1702017,1460296,1016014
3.1.3.4	1697718,1464474,1549600,1704013,785028,1297013
3.2.1.22	1292160

Supplementary Table 4. Annotated gene models in carotenoid biosynthesis

Gene name	Abbreviation	Protein ID
Geranylgeranyl pyrophosphate synthase	*GGPS*	1690735
Phytoene synthase	*PSY*	1470967
Phytoene desaturase	*PDS*	1479690
Zeta-carotene desaturase	*ZDS*	203596
Zeta-carotene isomerase	*ZISO*	1293889
Carotene isomerase	*CRTISO1*<br>*CRTISO2*<br>*CRTISO3*	1705674<br>868698<br>1645535
Lycopene epsilon-cyclase	*LCYE*	1636439
Lycopene beta-cyclase	*LCYB*	1281313
Cytochrome P450-type carotene hydroxylase (LUT5)	*CYP97A1*<br>*CYP97A2*	1295507<br>1701017
Beta-carotene hydroxylase	*CHYB*	1477468
Cytochrome P450-type carotene hydroxylase (LUT1)	*CYP97C*	1298651
Chlorophycean violaxanthin de-epoxidase	*CVDE*	211816
Zeaxanthin epoxidase	*ZEP1*	1519040
Neoxanthin synthase (ABA4)	*NSY*	1698300
Beta-ketolase	*BKT1*<br>*BKT2*<br>*BKT3*	1680266<br>1298489<br>1290392
CruP-type lycopene cyclase paralog	*CRUP*	604343

Supplementary References

1 Maul, J. E. *et al.* The Chlamydomonas reinhardtii plastid chromosome: islands of genes in a sea of repeats. *Plant Cell* **14**, 2659-2679, doi:10.1105/tpc.006155 (2002).

2 Starkenburg, S. R. *et al.* Draft nuclear genome, complete chloroplast genome, and complete mitochondrial genome for the biofuel/bioproduct feedstock species Scenedesmus obliquus strain DOE0152z. *Genome Announc* **5**, doi:10.1128/genomeA.00617-17 (2017).

3 Carreres, B. M. *et al.* Draft genome sequence of the oleaginous green alga Tetradesmus obliquus UTEX 393. *Genome Announc* **5**, doi:10.1128/genomeA.01449-16 (2017).

4 Carreres, B. M. *et al.* The diurnal transcriptional landscape of the microalga Tetradesmus obliquus. *Algal research* **40**, doi:10.1016/j.algal.2019.101477 (2019).

5 de Cambiaire, J. C., Otis, C., Lemieux, C. & Turmel, M. The complete chloroplast genome sequence of the chlorophycean green alga Scenedesmus obliquus reveals a compact gene organization and a biased distribution of genes on the two DNA strands. *BMC Evol Biol* **6**, 37, doi:10.1186/1471-2148-6-37 (2006).

6 Suzuki, S., Yamaguchi, H., Nakajima, N. & Kawachi, M. Raphidocelis subcapitata (=Pseudokirchneriella subcapitata) provides an insight into genome evolution and environmental adaptations in the Sphaeropleales. *Sci Rep* **8**, 8058, doi:10.1038/s41598-018-26331-6 (2018).

7 Bogen, C. *et al.* Reconstruction of the lipid metabolism for the microalga Monoraphidium neglectum from its genome sequence reveals characteristics suitable for biofuel production. *BMC Genomics* **14**, 926, doi:10.1186/1471-2164-14-926 (2013).

8 Roth, M. S. *et al.* Chromosome-level genome assembly and transcriptome of the green alga Chromochloris zofingiensis illuminates astaxanthin production. *Proc Natl Acad Sci U S A* **114**, E4296-E4305, doi:10.1073/pnas.1619928114 (2017).

9 Merchant, S. S. *et al.* The Chlamydomonas genome reveals the evolution of key animal and plant functions. *Science* **318**, 245-250, doi:10.1126/science.1143609 (2007).

10 Vahrenholz, C., Riemen, G., Pratje, E., Dujon, B. & Michaelis, G. Mitochondrial DNA of Chlamydomonas reinhardtii: the structure of the ends of the linear 15.8-kb genome suggests mechanisms for DNA replication. *Curr Genet* **24**, 241-247 (1993).
