# Supplementary material for: A multi-omic characterization of temperature stress in a halotolerant Scenedesmus strain for algal biotechnology
Source: Commun Biol. 2021 Mar 12;4:333. doi: 10.1038/s42003-021-01859-y (PMC7955037; doi:10.1038/s42003-021-01859-y)
Supplement: Supplementary file 3 — Description of Additional Supplementary Files [file 42003_2021_1859_MOESM3_ESM.pdf]

Description of Additional Supplementary Files

File Name: Supplementary Data File 1

Description: Screening and strain characterization data for Figures 1-2.

File Name: Supplementary Data File 2

Description: Species tree of Chlorophyta genomes in Newick format.

File Name: Supplementary Data File 3

Description: List of Pfam domains and ortholog gene families that are unique in *Scenedesmus* genomes.

File Name: Supplementary Data File 4

Description: Table of gene numbers for fatty acid biosynthesis, fatty acid elongation, and glycerolipid metabolism pathways in Chlorophyta genomes.

File Name: Supplementary Data File 5

Description: Table of growth according to optical density measurements shown in Figure 4.

File Name: Supplementary Data File 6

Description: Average fold change in abundance of triacylglycerol lipids (TAGs), monogalactosyldiacylglycerol lipids (MGDGs), and digalactosyldiacylglycerol lipids (DGDGs) in cold stress and heat stress samples relative to control samples at corresponding time points.

File Name: Supplementary Data File 7

Description: Average fold change in abundance of polar metabolites in cold stress and heat stress samples relative to control samples at corresponding time points.

File Name: Supplementary Data File 8

Description: Average gene expression (FPKM) for temperature stress and control samples over 24-hour sampling period.

File Name: Supplementary Data File 9

Description: First two principal components for principal component analysis of transcriptomics dataset in Figure 7.

File Name: Supplementary Data File 10

Description: Gene co-expression network calculated using weighted gene co-expression network analysis (WGCNA) in Figure 7.

File Name: Supplementary Data File 11

Description: Co-expression gene modules identified by weighted gene co-expression network analysis (WGCNA). GO enrichment analysis and annotations for genes in modules enriched with fatty acid biosynthesis genes are included.

File Name: Supplementary Data File 12

Description: Gene expression (FPKM) over time for genes involved in arginine metabolism in Figure 6.

File Name: Supplementary Data File 13

Description: Summary table for metabolite identification in positive and negative ion modes

File Name: Supplementary Data File 14

Description: Summary table for lipid identification including lipid standards, triacylglycerol lipids, monogalactosyldiacylglycerol lipids, and digalactosyldiacylglycerol lipids.
