# Supplementary material for: A multi-omic characterization of temperature stress in a halotolerant Scenedesmus strain for algal biotechnology
Source: Commun Biol. 2021 Mar 12;4:333. doi: 10.1038/s42003-021-01859-y (PMC7955037; doi:10.1038/s42003-021-01859-y)
Supplement: Supplementary file 18 — Reporting Summary [file 42003_2021_1859_MOESM18_ESM.pdf]

nature research

Corresponding author(s): Igor Grigoriev
Shawn Starkenburg

Last updated by author(s): 1/12/2021

Reporting Summary

Nature Research wishes to improve the reproducibility of the work that we publish. This form provides structure for consistency and transparency in reporting. For further information on Nature Research policies, see our Editorial Policies and the Editorial Policy Checklist.

Statistics

For all statistical analyses, confirm that the following items are present in the figure legend, table legend, main text, or Methods section.

n/a	Confirmed	
☐	☒	The exact sample size (*n*) for each experimental group/condition, given as a discrete number and unit of measurement
☐	☒	A statement on whether measurements were taken from distinct samples or whether the same sample was measured repeatedly
☐	☒	The statistical test(s) used AND whether they are one- or two-sided<br>*Only common tests should be described solely by name; describe more complex techniques in the Methods section.*
☒	☐	A description of all covariates tested
☐	☒	A description of any assumptions or corrections, such as tests of normality and adjustment for multiple comparisons
☐	☒	A full description of the statistical parameters including central tendency (e.g. means) or other basic estimates (e.g. regression coefficient) AND variation (e.g. standard deviation) or associated estimates of uncertainty (e.g. confidence intervals)
☐	☒	For null hypothesis testing, the test statistic (e.g. *F*, *t*, *r*) with confidence intervals, effect sizes, degrees of freedom and *P* value noted<br>*Give P values as exact values whenever suitable.*
☒	☐	For Bayesian analysis, information on the choice of priors and Markov chain Monte Carlo settings
☒	☐	For hierarchical and complex designs, identification of the appropriate level for tests and full reporting of outcomes
☒	☐	Estimates of effect sizes (e.g. Cohen's *d*, Pearson's *r*), indicating how they were calculated

Our web collection on statistics for biologists contains articles on many of the points above.

Software and code

Policy information about availability of computer code

Data collection	No software was used.
Data analysis	The following software programs were used, and further details are provided in the Methods section of the manuscript.<br>Genome: Falcon v 0.7.3, Celera, finisherSC, JGI annotation pipeline (BLAT, RepeatMasker, RepeatScout, Fgenesh, GeneWise, BlastX, SIgnalP, TMHMM, InterProScan), Braker v 1.9, Priam, PlantTFDB web server v 4.0, BUSCO v 4.0.5, GeSeq, Orthofinder v 2.3.8, RAxML v 8.2.7, FigTree v 1.4.4, CAFE v 4.2.1<br>Transcriptome: BBDuk, HISAT2 v 2.1.0, deepTools v 3.1, featureCounts, DESeq2 v 1.18.1<br>Metabolomics: Feature-Based Molecular Networking workflow (version release 18) using MZmine2

For manuscripts utilizing custom algorithms or software that are central to the research but not yet described in published literature, software must be made available to editors and reviewers. We strongly encourage code deposition in a community repository (e.g. GitHub). See the Nature Research guidelines for submitting code & software for further information.

Data

Policy information about availability of data

All manuscripts must include a data availability statement. This statement should provide the following information, where applicable:

- Accession codes, unique identifiers, or web links for publicly available datasets
- A list of figures that have associated raw data
- A description of any restrictions on data availability

The current genome assembly and annotations are publicly available at the JGI Genome Portal: https://phycocosm.jgi.doe.gov/Scesp_1. This Whole Genome Shotgun project has been deposited at DDBJ/ENA/GenBank under the accession JACERP000000000. The version described in this paper is version JACERP010000000. Genomics data have been deposited to SRA under the accession PRJNA407785. Transcriptomics data have been deposited to SRA under the

accession PRJNA672938.

Field-specific reporting

Please select the one below that is the best fit for your research. If you are not sure, read the appropriate sections before making your selection.

- [x] Life sciences
- [ ] Behavioural & social sciences
- [ ] Ecological, evolutionary & environmental sciences

For a reference copy of the document with all sections, see nature.com/documents/nr-reporting-summary-flat.pdf

Life sciences study design

All studies must disclose on these points even when the disclosure is negative.

Sample size	Compositional analysis used two biological replicates, as the range of variables tested and time points sampled were prioritized over additional replicates, reflecting previous methodologies. Samples for transcriptomic and metabolomic analyses used three biological replicates consistent with standard practice.
Data exclusions	No data was excluded from the analyses.
Replication	Experiments included multiple replicates.
Randomization	For LC/MSMS, sample injection order was randomized and an injection blank run between each sample. Otherwise, randomization was not performed, but sample controls were included.
Blinding	Blinding was not relevant to our study.

Reporting for specific materials, systems and methods

We require information from authors about some types of materials, experimental systems and methods used in many studies. Here, indicate whether each material, system or method listed is relevant to your study. If you are not sure if a list item applies to your research, read the appropriate section before selecting a response.

Materials & experimental systems

n/a	Involved in the study	
x		Antibodies
x		Eukaryotic cell lines
x		Palaeontology and archaeology
x		Animals and other organisms
x		Human research participants
x		Clinical data
x		Dual use research of concern

Methods

n/a	Involved in the study	
x		ChIP-seq
x		Flow cytometry
x		MRI-based neuroimaging
